# Preeclampsia by maternal reasons for immigration: a population-based study

**DOI:** 10.1186/s12884-018-2034-4

**Published:** 2018-10-26

**Authors:** Roy M Nilsen, Eline S Vik, Svein A Rasmussen, Rhonda Small, Dag Moster, Erica Schytt, Vigdis Aasheim

**Affiliations:** 1 Faculty of Health and Social Sciences, Western Norway University of Applied Sciences, Inndalsveien 28, 5063 Bergen, Norway; 2 Department of Clinical Science, University of Bergen, Bergen, Norway; 3 Judith Lumley Centre, School of Nursing & Midwifery, La Trobe University, Melbourne, Australia; 4 Reproductive Health, Department of Women’s and Children’s Health, Karolinska Institute, Stockholm, Sweden; 5 Department of Paediatrics, Haukeland University Hospital, Bergen, Norway; 6 Department of Global Public Health and Primary Care, University of Bergen, Bergen, Norway; 7 Centre for Clinical Research Dalarna, Uppsala University, Falun, Sweden

**Keywords:** Education, Family, Immigration, Labour, Preeclampsia, Pregnancy, Refugee

## Abstract

**Background:**

To investigate whether the occurrence of preeclampsia varied by maternal reasons for immigration.

**Methods:**

We included 1,287,270 singleton pregnancies (163,508 to immigrant women) in Norway during 1990–2013. Individual data were obtained through record linkage between the Medical Birth Registry of Norway and Statistics Norway. Analyses were performed for preeclampsia overall and in combination with preterm birth < 37 and < 34 weeks of gestation, referred to as preterm and very preterm preeclampsia. Odds ratios (ORs) with 95% confidence intervals (CIs) were estimated using logistic regression with robust standard errors, adjusted for relevant covariates, including maternal income and education.

**Results:**

Preeclampsia was reported in 3.5% of Norwegian women and 2.5% of immigrants. Compared with Norwegian women, the adjusted OR for preeclampsia was lowest in labour immigrants (adjusted OR 0.55 [95% CI 0.49–0.62]), followed by family immigrants (0.62 [0.59–0.65]), immigrant students (0.75 [0.65–0.86]), refugees (0.81 [0.75–0.88]), and immigrants from other Nordic countries (0.87 [0.80–0.94]). Compared with Norwegian women, labour immigrants also had lower adjusted odds of preterm and very preterm preeclampsia, whereas refugees had increased adjusted odds of preterm and very preterm preeclampsia (< 37 weeks: 1.18 [1.02–1.36], and < 34 weeks: 1.41 [1.15–1.72]).

**Conclusions:**

The occurrence of preeclampsia was lower overall in immigrants than in non-immigrants, but associations varied by maternal reasons for immigration. Maternity caregivers should pay increased attention to pregnant women with refugee backgrounds due to their excess odds of preterm preeclampsia.

**Electronic supplementary material:**

The online version of this article (10.1186/s12884-018-2034-4) contains supplementary material, which is available to authorized users.

## Background

People with an immigrant background constitute an increasing proportion of populations in many European countries. In 2017, the Norwegian population comprised 13.8% (~ 725,000) immigrants from 221 countries, with the largest proportions being from Europe, Asia, and Africa [1]. Major reasons for immigrating to Norway include employment, education, family reunion or establishment, as well as seeking refuge due to war and political conflicts [1, 2]. These immigration reasons may not be deterministically linked to countries of origin. Individuals from the same country may have different reasons for leaving, and individuals who arrive from very different countries may share immigration reasons.

Immigrants vary in health and disease compared with individuals born in the receiving countries [3, 4]. In terms of immigration reasons, individuals with a refugee background are considered a particularly vulnerable group and refugee background has been associated with several adverse outcomes in both pregnant and non-pregnant individuals [5, 6]. In particular, refugee women giving birth in receiving countries have been found to have higher risks of preterm birth [7], infant mortality and morbidity [8], and postpartum depression [9]. While these findings suggest the need for closer health monitoring of pregnant refugee women in receiving countries, knowledge of health disparities associated with other reasons such as employment, education, family reunion or establishment is lacking.

Increased knowledge of disparities in preeclampsia among immigrant pregnant women is of particular interest as preeclampsia is a leading cause of maternal and perinatal morbidity and mortality in many countries [10, 11]. Previous studies of preeclampsia in immigrant women in Western countries have shown that the occurrence of preeclampsia varies considerably by maternal birthplace [12–15]. A few studies have also reported that the occurrence of preeclampsia among immigrants increases with increasing length of residence in host countries [15, 16]. However, none of these studies performed analyses according to refugee status or other maternal reasons for immigration.

The main objective of the present study was to investigate whether the occurrence of preeclampsia varied by maternal reasons for immigration to Norway. We also explored whether the occurrence of preeclampsia varied with the length of residence for each immigration reason. Additionally, country-specific occurrences of preeclampsia in women from a number of countries were examined.

## Methods

### Data sources

Data were obtained through individual record linkage between the Medical Birth Registry of Norway (MBRN) and Statistics Norway. In brief, MBRN is a national health registry in which registration of all births after gestational week 16 has been compulsory in Norway since 1967 [17]. The registry comprises data on the mother’s health before and during pregnancy, on birth, and on the infant. Statistics Norway is the Norwegian institution for collection, processing, and dissemination of official statistics in Norway [18]. The collection of data relies on official registries and administrative data, including the National Registry, which contains data on individuals who either are, or have been resident in Norway [2].

### Preeclampsia

Preeclampsia for a given pregnancy was recorded once in the MBRN by a check box or open text coded according to the *International Statistical Classification of Diseases and Related Health Problems*, 8th revision for the years 1967–98 and 10th revision since 1999. The diagnostic criteria were defined as maternal blood pressure 140/90 mmHg or higher after gestational week 20 on at least 2 occasions, combined with proteinuria (+ 1 point increase on urinary dipstick) [19]. As an indicator of severity of preeclampsia, preeclampsia was analyzed overall as well as in combination with time point of birth. If a woman was diagnosed with preeclampsia and gave birth before gestational week 34 and 37, we defined her preeclampsia as very preterm preeclampsia and preterm preeclampsia, respectively. A validation study covering the years 1999–2010 showed that preeclampsia in MBRN corresponds well with medical records, although the sensitivity of the diagnosis may be somewhat low [20].

### Reasons for immigration

The collection of data regarding reasons for immigration to Norway has been described in detail elsewhere [2]. In brief, the information is obtained from the Norwegian Directorate of Immigration, which is the Norwegian authority for processing applications from foreign nationals who wish to visit or live in Norway. The immigration reason is recorded in connection with the first positive decision on the individual’s application for permission to stay in Norway which may or may not concur with the immigrant’s original motivation for immigration. Nordic citizens can move freely to Norway and therefore have no data regarding immigration reasons.

We used the variable derived by Statistics Norway, in which immigrants were allocated to one of five main categories of immigration reason: *refuge, family (reunion or establishment), labour, education, and unspecified reasons* [2]. For comparison, we also included immigrants from other Nordic countries (i.e., Sweden, Denmark, Finland, and Iceland) as a separate exposure group. Norwegian women constituted the reference group, and the group of women with unspecified reasons was excluded due to low numbers. Notably, data on immigration reasons were only available from 1990 onwards. Therefore, women who had received permission to stay in Norway before this time, but gave birth from 1990 onwards, were excluded from the study (see details below).

### Other variables

From the MBRN, we obtained data on year of birth, maternal age at birth, parity (0, 1, 2, 3, ≥4 previous births), marital status at birth (married/partner, single/widowed/other), chronic hypertension (yes, no) and pre-pregnancy diabetes (yes, no). Prenatal smoking and pre-pregnancy body mass index (kg/m^2^) were available from 1999 and 2008 onwards, respectively. From Statistics Norway, we obtained maternal data on country of birth (smaller countries with < 15 preeclampsia cases throughout the study period were grouped), maternal parents’ background (Norwegian born, foreign born), income (quartiles calculated for the whole study period) and educational level (no education, primary school, secondary school, university/college). The mother’s length of residence in years was calculated as year of childbirth in the MBRN minus the year of official permission to stay in Norway.

### Study sample

The study was restricted to include only ethnic Norwegian women (Norwegian-born with two Norwegian-born parents), other Nordic women, and first-generation immigrant women (foreign-born with two foreign-born parents) with a registered reason for immigration. Initially, there was a total 1,439,913 births during the period 1990–2013. We excluded 48,102 births due to multiple pregnancies and 389 pregnancies for which information on country of birth for the woman was missing. We additionally excluded 13,478 pregnancies of women who were born outside Norway but had two Norwegian born parents (including adoptees), 53,532 pregnancies of women who had one Norwegian and one foreign born parent (mixed-ethnic), and 6432 pregnancies of women who were born in Norway but had immigrant parents (second-generation immigrants). We further excluded 30,710 pregnancies for which information on immigration reasons was unavailable (permission to stay before 1990; *n* = 28,088), unspecified (*n* = 1886), or missing (*n* = 736), leaving 1,287,270 singleton pregnancies for analyses.

### Statistical analyses

To examine how the occurrence of preeclampsia varied by immigration reason we used binary logistic regression models. Immigration reason was incorporated in the models as a categorical variable with Norwegian women as the reference group. We calculated both crude and adjusted odds ratios (ORs) with 95% confidence intervals (CIs). Adjustment variables were year of birth, maternal age at birth, parity, marital status at birth, chronic hypertension, pre-pregnancy diabetes, maternal income and education. Year of birth and maternal age at birth were included in the regression models as polynomial quadratic terms. To account for dependency among births by the same mother, we used robust standard errors that allowed for within-mother clustering.

We also estimated the incidence of preeclampsia in relation to length of residence for each immigration group. The length of residence was modeled as a continuous exposure using generalized additive logistic regression models, allowing for nonlinear relationships. The predicted incidences are presented in graphical format for primiparous and multiparous women, with adjustment for the same variable as previously. To test if preeclampsia incidence trajectories differed across immigration groups, we performed a likelihood ratio test by comparing the log-likelihood for a model with and without the interaction between length of residence and immigration reasons. Due to limited data within subgroups, estimations were performed only for preeclampsia overall and not for preterm and very preterm preeclampsia.

All statistical analyses were performed by using R 3.4.1 software for Windows [21]. Missing data on maternal income and education (shown in footnotes of Table 1) were assumed to be missing at random and were replaced by using a multiple imputation technique [22]. Five imputed datasets were created using the predictive mean matching algorithm. The imputation model included the same adjustment variables as before, as well as data on immigration reason, maternal country of birth and preeclampsia.

**Table 1:** Sample characteristics by reasons for immigration to Norway, 1990–2013

Characteristics	Norwegian women (non-immigrants)	Nordic immigrant women	Reasons for immigration			
			Refuge	Family	Labour	Education
No. of women	1,123,762	22,594	29,422	89,523	13,618	8351
Period of birth (%)						
1990–1993	18.6	12.5	5.2	3.4	0.4	1.0
1994–1998	22.4	18.1	11.4	11.1	2.2	6.0
1999–2003	20.4	21.9	19.5	20.2	6.6	12.7
2004–2008	19.7	21.9	27.7	28.8	18.0	26.6
2009–2013	18.9	25.6	36.2	36.4	72.8	53.6
Maternal age at birth, years (mean ± SD)	29.0 ± 5.1	30.6 ± 4.8	28.6 ± 5.6	29.1 ± 5.4	30.2 ± 4.7	30.1 ± 4.0
Parity (%)						
Primiparous	41.2	45.6	31.5	41.7	60.8	58.7
Multiparous	58.8	54.4	68.5	58.3	39.2	41.3
Single/widowed/other (%)	8.2	5.0	19.3	5.3	4.0	5.5
Maternal income, NOK per 1000 (quartiles)^a^						
< 125.0	24.7	16.3	37.3	35.3	13.0	24.3
125.0–195.5	25.6	20.7	18.7	20.2	12.5	12.0
195.6–287.4	25.0	28.1	23.0	24.0	28.1	22.0
≥ 287.5	24.7	34.8	21.0	20.5	46.3	41.6
Maternal educational level (%)^b^						
No education	0.0	0.4	7.6	2.8	0.3	0.2
Primary school	21.6	10.6	50.5	36.0	7.6	12.5
Secondary school	38.5	31.2	25.5	26.8	21.9	16.5
University/college	39.9	57.8	16.5	34.3	70.2	70.8
Smoking early in pregnancy (%)^c^	18.3	11.6	7.5	5.7	8.5	2.8
Pre-pregnancy BMI, kg/m^2^ (mean ± SD)^d^	24.6 ± 4.9	24.0 ± 4.5	24.7 ± 4.8	23.5 ± 4.4	23.0 ± 4.0	22.8 ± 3.9
Chronic hypertension (%)	0.5	0.6	0.4	0.3	0.6	0.5
Pre-pregnancy diabetes (%)	0.4	0.4	0.6	0.6	0.4	0.4
Length of residence, years (mean ± SD)^e^		7.0 ± 6.1	5.5 ± 5.2	4.1 ± 4.0	3.2 ± 2.7	6.1 ± 3.8
Maternal age at arriving, years (mean ± SD)^e^		23.6 ± 6.6	23.1 ± 6.9	25.0 ± 5.8	27.0 ± 4.3	24.0 ± 3.6

*SD* standard deviation, *NOK* Norwegian kroner, *BMI* body mass index

^a^Information on income was missing for 94,234 (8.4%) among non-immigrants and 70,977 (43.4%) among immigrants

^b^Information on education was missing for 2229 (0.2%) among non-immigrants and 45,779 (28.0%) among immigrants

^c^Information on smoking (1999–2013) was missing for 96,763 (14.6%) among non-immigrants and 31,306 (22.7%) among immigrants

^d^Information on body mass index (2008–2013) was missing for 137,574 (53.5%) among non-immigrants and 39,014 (53.1%) among immigrants

^e^Excluded were 1303 (0.8%) immigrant women who were registered with births before receiving permission to stay in Norway

## Results

The number of immigrants and non-immigrants giving birth in the current sample was 163,508 (13%) and 1,123,762 (87%), respectively. The number of registered maternal birth countries was 186.

Among immigrant births, 18% (*n* = 29,422) were to women coming as refugees, 55% (*n* = 89,523) to family immigrants, 8% (*n* = 13,618) to labour immigrants, 5% (*n* = 8351) to immigrant students, and 14% (*n* = 22,594) were to Nordic immigrant women. There was a steady increase in the number of immigrants in all groups from 1990 to 2013, although labour immigrants increased more rapidly during the most recent period than did other groups (Table 1). The countries dominating each immigration reason are shown in Additional file 1.

Immigrants differed on several sample characteristics (Table 1). Refugees were younger when giving birth, had a higher parity, and were more often single than others. They also had lower income and lower education than that of others. In contrast, women coming for employment or education had lower parity, lower mean body mass index and higher education and income than others. Refugees and family immigrants had less chronic hypertension than Norwegians. Norwegian and Nordic immigrant women had the highest smoking prevalence (18% and 12%, respectively).

There was considerable variation in the mean length of residence from arrival to childbirth across immigration groups (Table 1). Nordic immigrant women and those coming for education had nearly twice the residence length (mean 7.0 and 6.1 years, respectively) compared to labour and family immigrants (mean 3.2 and 4.1 years, respectively).

**Fig. 1:**
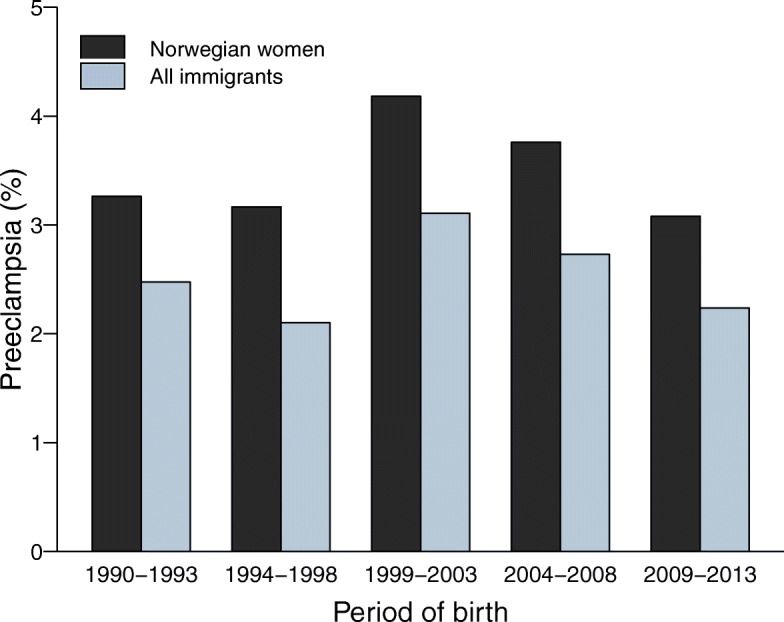
Incidence of preeclampsia in Norwegian and immigrant women by period of birth in Norway, 1990–2013

Overall, preeclampsia was reported in 3.5% (*n* = 39,251) of non-immigrants and in 2.5% (*n* = 4133) of immigrants. The corresponding incidences in nulliparous women were 5.2% (*n* = 24,043) and 3.5% (*n* = 2461), respectively. The lower incidence of preeclampsia in immigrant women remained constant throughout the study period (Fig. 1). However, there was considerable variation across women’s countries of birth with immigrants from several countries having adjusted odds of preeclampsia higher than that seen in Norwegian women (Additional file 2).

Table 2 shows the incidence and the corresponding OR for preeclampsia in relation to immigration reasons. All immigration reasons were associated with lower preeclampsia incidence. Relative to Norwegian women, the adjusted OR for preeclampsia was lowest in labour immigrants (adjusted OR 0.55 [95% CI 0.49–0.62]), followed by family immigrants (0.62 [0.59–0.65]), immigrant students (0.75 [0.65–0.86]), refugees (0.81 [0.75–0.88]), and Nordic immigrant women (0.87 [0.80–0.94]).

**Table 2:** Odds ratio for preeclampsia by reasons for immigration to Norway, 1990–2013

Reasons for immigration	No. of women	With outcome, No. (%)	Crude OR [95% CI]	Adjusted OR [95% CI]^a^	Adjusted OR [95% CI]^b^
Preeclampsia (all cases)					
Norwegian women (non-immigrants)^c^	1,123,762	39,251 (3.5)	1	1	1
Nordic immigrant women	22,594	723 (3.2)	0.91 [0.84–0.99]	0.86 [0.79–0.93]	0.87 [0.80–0.94]
Refuge	29,422	800 (2.7)	0.77 [0.71–0.84]	0.87 [0.81–0.95]	0.81 [0.75–0.88]
Family	89,523	2074 (2.3)	0.66 [0.62–0.69]	0.65 [0.62–0.69]	0.62 [0.59–0.65]
Labour	13,618	293 (2.2)	0.61 [0.54–0.68]	0.56 [0.49–0.63]	0.55 [0.49–0.62]
Education	8351	243 (2.9)	0.83 [0.72–0.95]	0.75 [0.66–0.86]	0.75 [0.65–0.86]
Preterm preeclampsia (< 37 weeks)^d^					
Norwegian women (non-immigrants)^c^	1,077,269	8431 (0.8)	1	1	1
Nordic immigrant women	21,948	157 (0.7)	0.91 [0.77–1.08]	0.84 [0.71–1.00]	0.86 [0.72–1.01]
Refuge	28,304	257 (0.9)	1.16 [1.01–1.34]	1.28 [1.11–1.47]	1.18 [1.02–1.36]
Family	87,571	647 (0.7)	0.94 [0.87–1.03]	0.93 [0.85–1.02]	0.88 [0.80–0.96]
Labour	13,513	68 (0.5)	0.64 [0.50–0.81]	0.58 [0.45–0.73]	0.58 [0.45–0.74]
Education	8254	64 (0.8)	0.99 [0.76–1.29]	0.89 [0.68–1.16]	0.89 [0.69–1.16]
Very preterm preeclampsia (< 34 weeks)^d^					
Norwegian women (non-immigrants)^c^	1,077,269	3480 (0.3)	1	1	1
Nordic immigrant women	21,948	57 (0.3)	0.80 [0.61–1.06]	0.73 [0.56–0.97]	0.75 [0.57–0.98]
Refuge	28,304	132 (0.5)	1.45 [1.19–1.75]	1.56 [1.28–1.89]	1.41 [1.15–1.72]
Family	87,571	280 (0.3)	0.99 [0.87–1.13]	0.98 [0.86–1.12]	0.91 [0.80–1.05]
Labour	13,513	32 (0.2)	0.73 [0.52–1.04]	0.64 [0.45–0.92]	0.65 [0.46–0.93]
Education	8254	30 (0.4)	1.13 [0.78–1.63]	0.99 [0.68–1.44]	1.00 [0.69–1.46]

*OR* odds ratio, *CI* confidence interval

^a^Adjusted for year of birth, maternal age at birth, parity, marital status at birth, chronic hypertension, and pre-pregnancy diabetes

^b^Additional adjustments for maternal income and education

^c^Reference category

^d^Excluded were 50,411 pregnancies (1736 with and 48,675 without preeclampsia) due to missing data on gestational age

Additional adjustment for maternal prenatal smoking (1999–2013; *n* = 673,286) had essentially no impact on the estimates, compared with the adjusted OR (excluding smoking) in the same period (data not shown).

Women coming for employment also had lower adjusted odds if preeclampsia occurred preterm or very preterm (Table 2). In contrast, refugees had excess adjusted odds of preterm and very preterm preeclampsia (< 37 weeks: 1.18 [1.02–1.36] and < 34 weeks: 1.41 [1.15–1.72]). For other groups, adjusted ORs for preterm or very preterm preeclampsia were either attenuated (family and education) or remained essentially unchanged (Nordic immigrant women).

Among immigrant women for which information on immigration reasons was unavailable, unspecified or missing, the incidence of preeclampsia was 2.7% (833/30,710).

**Fig. 2:**
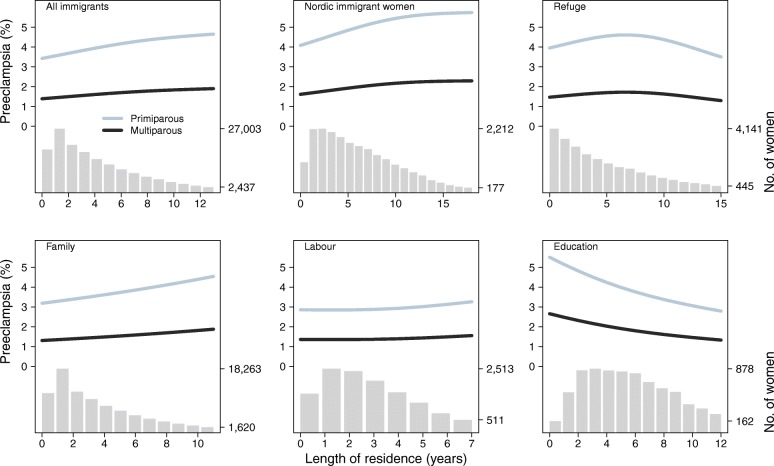
Estimated adjusted incidence of preeclampsia by length of residence for various immigration reasons in Norway, 1990–2013. The incidences were estimated for primiparous and multiparous immigrant women by using generalized additive logistic regression models, adjusted for year of birth, maternal age at birth, parity, marital status at birth, chronic hypertension, pre-pregnancy diabetes, maternal income and education. The incidence trajectories for each immigration group are shown for secondary school and third income quartile and at the means of the other covariates (see Table 1). Due to small numbers, lengths of residence above the 95th percentile of the distributions were excluded. The distribution of length of residence is shown on the x-axis as frequency bars (highest and lowest frequencies are shown on right vertical axis)

Figure 2 shows the estimated adjusted incidence of preeclampsia according to length of residence for each immigration group. The adjusted incidence of preeclampsia for Nordic immigrant women and family immigrants appeared to increase with increasing length of residence. For labour immigrants and refugees, the adjusted incidence of preeclampsia remained essentially constant over time, while there was a decline in the adjusted incidence for students. The *P* for interaction across immigration groups was estimated to be < 0.001.

## Discussion

We found that the occurrence of preeclampsia was generally lower in immigrants than in non-immigrants, but that the disparity varied by reasons for immigration and severity of preeclampsia. Particularly, labour immigrants had a substantially lower OR for preeclampsia overall as well as for preterm and very preterm preeclampsia. In contrast, refugees had an excess OR for preterm and very preterm preeclampsia. Furthermore, there was an increase in the adjusted incidence of preeclampsia with the length of residence for Nordic immigrant women and family immigrants, but not for the other immigrant groups.

As far as we are aware, this represents the first study investigating how the occurrence of preeclampsia varies by maternal reasons for immigration. Strengths of our study include the large sample size, the standardized collection of data on both preeclampsia and migrant-related variables and the comprehensive adjustment for covariates in regression analyses.

The results of our study are not generalizable to all women giving birth in Norway. Particularly, we excluded second-generation immigrants, mixed-ethnic mothers, and adoptees, as these were neither ethnic Norwegian women nor non-Nordic immigrants with an immigration reason. Additionally, as the composition of immigrant groups in Norway may differ from that in other countries, our results may not be completely generalizable to other countries. Additional file 2 shows the largest groups dominating each immigration reason in our study (i.e., countries covering at least 50% of each group).

Pre-pregnancy body mass index is associated with both preeclampsia and immigrant background, but was not adjusted for in the analyses due to the variable’s limited registration history in the MBRN (registered from 2008). Adjusting for body mass index would probably have attenuated the difference between Norwegian women and labour immigrants and immigrant students, because these immigrant groups were leaner than others. However, the adjustment of several demographic and socioeconomic variables might have compensated for some of this variation as demographic and socioeconomic variables are related to both obesity and preeclampsia.

The estimated incidences of preeclampsia for specific maternal countries of birth in our study were somewhat lower than those estimated in a previous Norwegian study including women from eight birth countries for the period 1986–2005 [15]. Variation in preeclampsia incidences between studies may reflect different study periods, different inclusion criteria (e.g., multiple births vs singletons), or variation in sample characteristics and immigrant groups over the study periods. Nonetheless, we found that Vietnamese and Chinese women had the lowest incidence of preeclampsia while women from Bangladesh and several countries from sub-Saharan Africa had among the highest (see Fig. 2). This partly agrees with the Norwegian study and a recent study from Canada on preterm preeclampsia [12, 15].

Consistent with previous studies [23], we found that immigrants had an overall lower incidence of preeclampsia than non-immigrants. In our sample, results were particularly strong for labour immigrants, but family immigrants, immigrant students, refugees, and immigrants from other Nordic countries also exhibited lower overall incidence of preeclampsia compared with Norwegian women. Our results regarding a lower overall incidence of preeclampsia in refugees agree with two recent studies comparing refugee pregnant women with Turkish and Canadian-born pregnant women [24, 25]. A comparison of results with studies concerning other immigration reasons was difficult due to the scarce literature.

The phenomenon that immigrants exhibit lower disease rates than the host population has been reported for numerous health outcomes. It is usually explained in terms of “the healthy immigrant effect”, i.e., people who immigrate are a selected group and on average healthier than the population they move to [26]. Indeed, immigrants tended to be healthy in our study; in addition to having lower incidence of preeclampsia, we found that several immigrant groups were less often smokers, had a lower mean body mass index, and had less chronic hypertension than Norwegian mothers (see Table 1).

We did not find consistent lower incidences for preterm and very preterm preeclampsia among immigrants. Rather, in comparison with Norwegian women, a higher incidence for preterm and very preterm preeclampsia was found for refugees, and no increased or reduced incidence was observed for immigrant students. Our finding that refugee women were more vulnerable to preterm preeclampsia appears to be supported by previous literature. Particularly, refugees suffer more frequently than others from mental health problems, such as posttraumatic stress disorders, depression and schizophrenia [27, 28]. These disorders have been associated with increased risk of both preeclampsia and preterm birth in pregnant women [29–33].

We found an overall increase in the adjusted incidence of preeclampsia with the length of residence (see Fig. 2). This confirms previous findings and may partly be due to a gradual change in risk factors for preeclampsia after immigration [15, 16]. For instance, dietary changes over time may contribute to increased risk of obesity, diabetes, and cardiovascular disease [34]. Notably, in the present analysis, we did not find positive associations between length of residence and preeclampsia among refugees, students and labour immigrants. This may suggest that positive and negative determinants of preeclampsia in these groups were unchanged or cancelled each other out over the study period.

It could be argued that the information about an individual’s reason for immigration merely reflects their country of birth, and that immigration reason is therefore superfluous. Although this may be partly true for refugees coming from conflict-laden countries, such an assumption would be problematic for the other immigration reasons, such as labour, family or education, which are less country-specific. Additionally, using country as an indicator for immigration reasons could lead to biased conclusions, as immigration reasons from any one country can, and do, vary between individuals, or, in some instances, change over time depending on a country’s economic and political situation. In light of this, we believe that immigration reasons attributed individually to immigrant woman are likely to be a valuable indicator for investigating perinatal health among immigrants.

## Conclusions

In this large population-based study, we used immigration reason as a health indicator for identifying immigrant women with high and low occurrence of preeclampsia in Norway. Our study suggests that maternity caregivers should pay increased attention to pregnant women of refugee background due to their higher odds of preterm preeclampsia. Among Nordic immigrant women and family immigrants, the aim should be to support the maintenance of healthy behaviors in these women to keep the occurrence low after immigration. Labour immigrants and immigrant students appear to be the least vulnerable groups, as the occurrence of preeclampsia in these groups was unchanged or decreased over time. However, from a public health perspective continued monitoring of all groups is necessary to detect potential variation in preeclampsia over time.

## Additional files

Additional file 1: Reasons for immigration to Norway by maternal country of birth, 1990–2013 (DOCX 16 kb)

Additional file 2: Incidence and adjusted odds ratios of preeclampsia by maternal country of birth in Norway, 1990–2013. Odds ratios and 95% confidence intervals were estimated by using logistic regression models, adjusted for year of birth, maternal age at birth, parity, marital status at birth, chronic hypertension, pre-pregnancy diabetes, and maternal income, and education. The reference group was Norwegian women (i.e., non-immigrants) and women from countries with < 15 preeclampsia cases throughout the study period were grouped. *OR* odds ratio, *CI* confidence interval (PDF 16 kb)

## Abbreviations

CI: Confidence interval
MBRN: Medical Birth Registry of Norway
OR: Odds ratio
