# Supplementary material for: Preeclampsia by maternal reasons for immigration: a population-based study
Source: BMC Pregnancy Childbirth. 2018 Oct 26;18:423. doi: 10.1186/s12884-018-2034-4 (PMC6204029; doi:10.1186/s12884-018-2034-4)
Supplement: Supplementary file 1 — Reasons for immigration to Norway by maternal country of birth, 1990–2013 (DOCX 16 kb) [file 12884_2018_2034_MOESM1_ESM.docx]

| Reasons for immigration ^a^ | |  | No. (%) | |
| --- | --- | --- | --- | --- |
| Refuge | |  | *29,422 (100)* |  |
|  | Somalia |  | 7,471 (25.4) | |
|  | Former Yugoslavia |  | 6,553 (22.3) | |
|  | Iraq |  | 2,538 (8.6) | |
|  | Other countries |  | 12,860 (43.7) | |
| Family | |  | *89,523 (100)* |  |
|  | Pakistan |  | 7,430 (8.3) | |
|  | Iraq |  | 6,480 (7.2) | |
|  | Thailand |  | 5,628 (6.3) | |
|  | Poland |  | 5,144 (5.7) | |
|  | Somalia |  | 4,945 (5.5) | |
|  | Sri Lanka |  | 4,336 (4.8) | |
|  | Philippines |  | 4,321 (4.8) | |
|  | Former Yugoslavia |  | 4,129 (4.6) | |
|  | Turkey |  | 3,846 (4.3) | |
|  | Other countries |  | 43,264 (48.3) | |
| Labour | |  | *13,618 (100)* |  |
|  | Poland |  | 3,432 (25.2) | |
|  | Germany |  | 1,943 (14.3) | |
|  | Lithuania |  | 1,840 (13.5) | |
|  | Other countries |  | 6,403 (47.0) | |
| Education | |  | *8,351 (100)* |  |
|  | Philippines |  | 1,349 (16.2) | |
|  | Russia |  | 819 (9.8) | |
|  | Germany |  | 491 (5.9) | |
|  | Lithuania |  | 408 (4.9) | |
|  | Poland |  | 401 (4.8) | |
|  | China |  | 400 (4.8) | |
|  | Ukraine |  | 352 (4.2) | |
|  | Other countries |  | 4,131(49.5) | |

^a^ Only the largest countries covering at least 50% of each immigration reason are listed
