# Supplementary figures and images for: Preeclampsia by maternal reasons for immigration: a population-based study
Source: BMC Pregnancy Childbirth. 2018 Oct 26;18:423. doi: 10.1186/s12884-018-2034-4 (PMC6204029; doi:10.1186/s12884-018-2034-4)

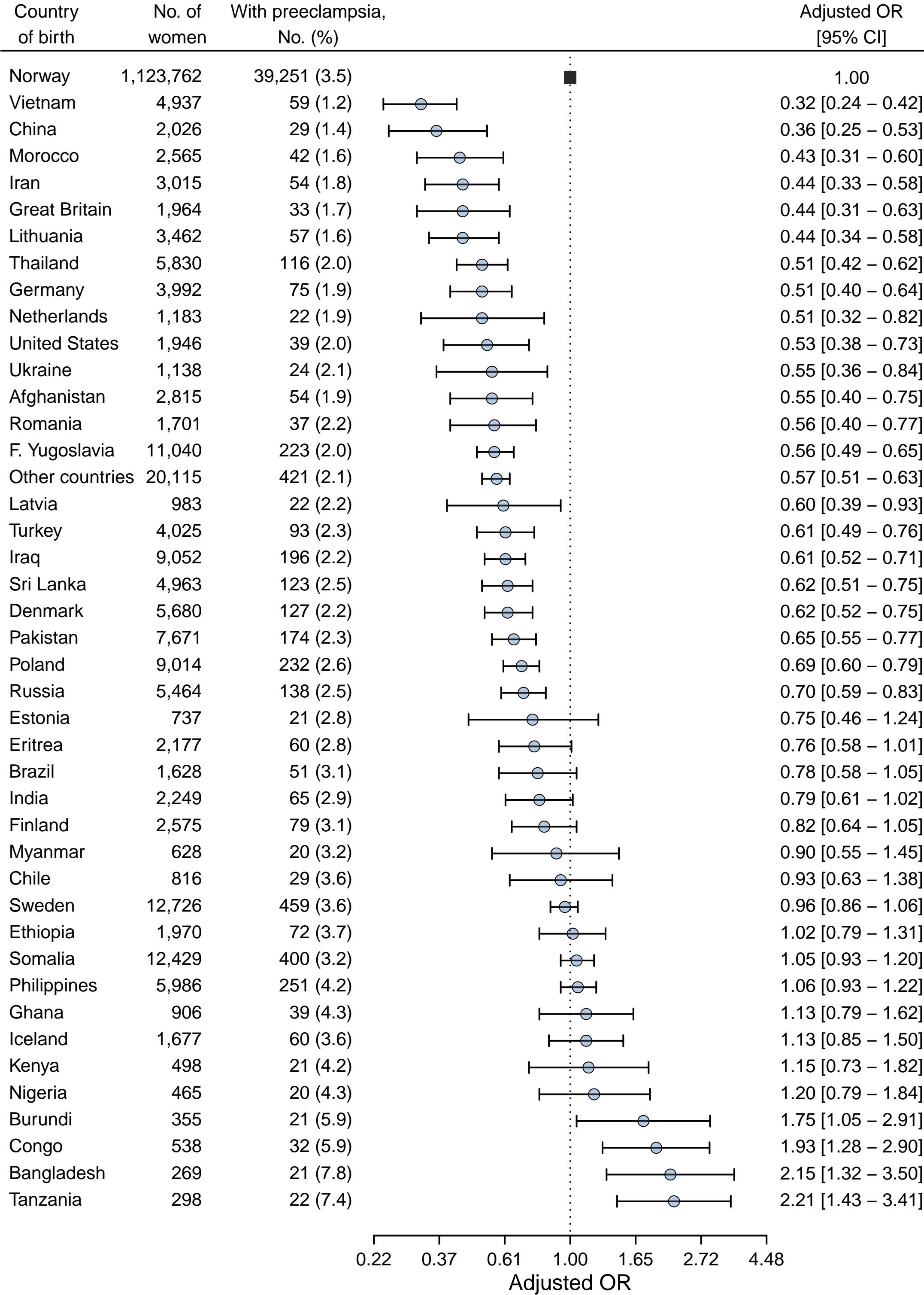

Supplement: Supplementary file 2 — Incidence and adjusted odds ratios of preeclampsia by maternal country of birth in Norway, 1990–2013. Odds ratios and 95% confidence intervals were estimated by using logistic regression models, adjusted for year of birth, maternal age at birth, parity, marital status at birth, chronic hypertension, pre-pregnancy diabetes, and maternal income, and education. The reference group was Norwegian women (i.e., non-immigrants) and women from countries with < 15 preeclampsia cases throughout the study period were grouped. OR odds ratio, CI confidence interval (PDF 16 kb) [file 12884_2018_2034_MOESM2_ESM.pdf]
